# Control of excitatory synaptic transmission by capsaicin is unaltered in TRPV_1_ vanilloid receptor knockout mice

**DOI:** 10.1016/j.neuint.2007.06.008

**Published:** 2008-01

**Authors:** Felix Benninger, Tamás F. Freund, Norbert Hájos

**Affiliations:** Department of Cellular and Network Neurobiology, Institute of Experimental Medicine, Hungarian Academy of Sciences, Budapest H-1450, Hungary

**Keywords:** Brain slices, Glutamate, Transmitter release, Dentate gyrus, Granule cell, Excitatory synapses

## Abstract

Several studies have shown that capsaicin could effectively regulate excitatory synaptic transmission in the central nervous system, but the assumption that this effect is mediated by TRPV_1_ vanilloid receptors (TRPV_1_Rs) has not been tested directly. To provide direct evidence, we compared the effect of capsaicin on excitatory synapses in wild type mice and TRPV_1_R knockouts. Using whole-cell patch-clamp techniques, excitatory postsynaptic currents (EPSCs) were recorded in granule cells of the dentate gyrus. First, we investigated the effect of capsaicin on EPSCs evoked by focal stimulation of fibers in the stratum moleculare. Bath application of 10 μM capsaicin reduced the amplitude of evoked EPSCs both in wild type and TRPV_1_R knockout animals to a similar extent. Treatment of the slices with the TRPV_1_R antagonist capsazepine (10 μM) alone, or together with the agonist capsaicin, also caused a decrease in the EPSC amplitude both in wild type and TRPV_1_R knockout animals. Both drugs appeared to affect the efficacy of excitatory synapses at presynaptic sites, since a significant increase was observed in paired-pulse ratio of EPSC amplitude after drug treatment. Next we examined the effect of capsaicin on spontaneously occurring EPSCs. This prototypic vanilloid ligand increased the frequency of events without changing their amplitude in wild type mice. Similar enhancement in the frequency without altering the amplitude of spontaneous EPSCs was observed in TRPV_1_R knockout mice.

These data strongly argue against the hypothesis that capsaicin modulates excitatory synaptic transmission by activating TRPV_1_Rs, at least in the hippocampal network.

## 1 Introduction

Capsaicin, the pungent ingredient of red peppers, severely affects pain sensation, inflammation or hyperalgesia. Systematic studies aiming to elucidate the effects of capsaicin revealed that this alkaloid primarily targets sensory fibers of the C type, where it activates a member of the transient receptor potential (TRP) channels, TRPV_1_ vanilloid receptors (TRPV_1_Rs) (Szallasi and Blumberg, 1999; Szolcsanyi, 2004). These receptors, cloned by Caterina et al. (1997), are non-selective cation channels gated by heat, low pH or endogenous ligands, such as anandamide (Tominaga et al., 1998; Zygmunt et al., 1999; Caterina et al., 1999; Smart et al., 2000). In addition, capsaicin could also affect the operation of both voltage-gated sodium and calcium channels (Balla et al., 2001; Lundbaek et al., 2005; Kofalvi et al., 2006), indicating that some of the capsaicin effects might not be linked to TRPV_1_Rs.

In contrast to the well-established function of TRPV_1_Rs in the periphery, its role is much less obvious in the central nervous system. Using autoradiography or immunohistochemistry, TRPV_1_Rs were shown to be present in several brain regions, including cortical structures (Acs et al., 1996; Mezey et al., 2000; Roberts et al., 2004; Toth et al., 2005; Cristino et al., 2006). Importantly, the specificity of signals in two of these reports has been confirmed in TRPV_1_R knockout mice (Roberts et al., 2004; Cristino et al., 2006), strongly arguing for the existence of TRPV_1_Rs in the CNS, yet their subcellular (synaptic or extrasynaptic) localization remains to be determined by high resolution electron microscopy. The functional role of TRPV_1_Rs in distinct brain regions was addressed by electrophysiological experiments. In the hippocampus, for example, an increase in paired-pulse depression of field potentials after application of capsaicin or anandamide has been noticed, and this effect was found to be sensitive to TRPV_1_R antagonists (Al-Hayani et al., 2001; Huang et al., 2002). In addition to the investigation of capsaicin effects on field potentials, other studies have examined the activation of putative TRPV_1_Rs on synaptic transmission more directly. These studies have found that in distinct parts of the brain, glutamatergic, but not GABAegic synaptic communication could be controlled by capsaicin, an effect that was also antagonized by TRPV_1_R antagonists (Sasamura et al., 1998; Hájos and Freund, 2002; Marinelli et al., 2002, 2003; Xing and Li, 2007). Interestingly, excitatory postsynaptic currents (EPSCs) evoked by electrical stimulation were found to be depressed after application of capsaicin, whereas the same treatment significantly increased the occurrence of spontaneous EPSCs without affecting their amplitude.

To reveal whether the effect of capsaicin on synaptic glutamate release is indeed mediated by TRPV_1_Rs, we investigated the properties of EPSCs in dentate granule cells after bath application of this prototypic vanilloid ligand both in wild type and TRPV_1_R knockout mice.

## 2 Experimental procedures

**Fig. 1:**
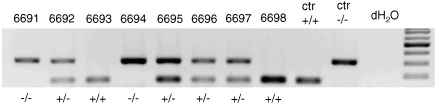
Results of genotyping a litter. Presence of a 150 bp length fragment indicates wild type allele, 300 bp PCR fragment shows targeted allele. TRPV_1_Rs (+/+, wild type;+/−, heterozygote; −/−, knockout). Controls (ctr) for +/+ and −/− are also indicated.

Experiments were carried out according to the guidelines of the institutional ethical code and the Hungarian Act of Animal Care and Experimentation (1998. XXVIII. section 243/1998). Wild type and TRPV_1_R knockout mice of both sexes (20–77 days old, C57BL/6J strain) (Davis et al., 2000) were used. Mouse genotyping was performed on tail DNA. Neo PCR primer sequences were the following: NeoF 5′-CCGGCCGCTTGGGTGGAGAGG and NeoR 5′-products on 300 bp (targeted allele), and TRPV_1_Rs (VRF1 5′-CATGGCCAGTGAGAACACCATGG and VRR2 5′-AGCCTTTTGTTCTTGGCTTCTCCT) products on 150 bp (wild type allele). Amplification reactions were carried out in 25 μl total volume with presence of 1% dimethyl-formamide, 0.2 μM primer each, 0.2 μM dNTP, 1.5 mM MgCl_2_ (93 °C for 15 s, 58 °C for 15 s, 72 °C for 45 s, for 30 cycles). Amplification products were analyzed by agarose gel electrophoresis on 1.2% agarose gels. An example for the results of genotyping of a litter is shown in Fig. 1.

The animals were deeply anaesthetized with isoflurane followed by decapitation. After opening the skull, the brain was quickly removed and immersed into ice-cold cutting solution containing (in mM: NaCl 126, KCl 2.5, NaHCO_3_ 26, CaCl_2_ 0.5, MgCl_2_ 5, NaH_2_PO_4_ 1.25, glucose 10) bubbled with 95% O_2_/5% CO_2_ (carbogen gas). Thick horizontal slices (300–350 μm from mice) were prepared using a Leica VT1000S Vibratome. The slices were stored in an interface type chamber containing ACSF (in mM: 126 NaCl, 2.5 KCl, 26 NaHCO_3_, 2 CaCl_2_, 2 MgCl_2_, 1.25 NaH_2_PO_4_, and 10 glucose) at room temperature for at least 1 h before recording.

Whole-cell patch-clamp recordings were obtained at 34–36 °C from granule cells in the dentate gyrus visualized by infrared videomicroscopy (Versascope, Marton Electronics, Canoga Park, CA). Patch electrodes were pulled from borosilicate glass capillaries with an inner filament (1.5 mm o.d., 1.12 mm i.d.; Hilgenberg, Germany) using a Sutter P-87 puller. Electrodes (∼3–6 MΩ) were filled with a solution containing (in mM) 80 CsCl, 60 Cs-gluconate, 3 NaCl, 1 MgCl_2_, 10 HEPES, 2 Mg-ATP, and 5 QX-314 (pH 7.2–7.3 adjusted with CsOH; osmolarity 275–290 mOsm). Excitatory postsynaptic currents (EPSCs) were recorded at a holding potential of −65 mV. Slices were perfused with ACSF containing 70–100 μM picrotoxin to block inhibitory neurotransmission. The solution was bubbled with carbogen gas at room temperature and perfused at a flow rate of 2–3 ml/min in a submerged type chamber. To evoke EPSCs, the stimulating electrode was placed in the stratum moleculare of the dentate gyrus. Pairs of electrical stimuli separated by 50 ms were delivered via a theta glass pipette (Sutter Instrument Company, Novato, CA) filled with ACSF at 0.1 Hz using a Supertech timer and isolator (Supertech Ltd., Pécs, Hungary, http://www.superte.ch). Access resistances (between 4 and 18 MΩ, compensated 65–70%) were frequently monitored and remained constant (±20%) during the period of analysis. Signals were recorded with an Axopatch 200B (Molecular Devices, Sunnyvale, CA), filtered at 2 kHz, digitized at 6 kHz (National Instruments PCI-6024E A/D board, Austin, TX), and analyzed off-line with the EVAN program (courtesy of Prof. I. Mody, UCLA, CA).

The drugs were perfused until the maximal effect was reached (usually 3–4 min). The effect of drugs on evoked EPSCs was calculated as follows: control EPSC amplitudes in a 2–3 min time window were compared to those measured after 5–6 min drug application for the same period of time. Only those experiments were included that had stable amplitudes at least for 10 min before drug application. The paired-pulse ratio was calculated from the mean amplitude of the second EPSCs divided by the mean amplitude of the first EPSCs. For spontaneously occurring EPSCs, the amplitude and the inter-event interval for individual events were calculated and medians of their distributions were compared before and after 5 min of capsaicin application. After each experiment, the tubing made of Teflon was washed with ethanol for 10 min and with ACSF for 15 min. For comparison of data, Wilcoxon matched pairs test or Mann–Whitney *U*-test were used in STATISTICA 6.1 (Statsoft, Inc., Tulsa, OK). Data are presented as mean ±S.E.M.

Picrotoxin was purchased from Sigma–Aldrich, while (*E*)-capsaicin and capsazepine were obtained from Tocris. Both drugs were dissolved in DMSO giving a 100 mM stock solution, which were stored at 4 °C.

## 3 Results

**Fig. 2:**
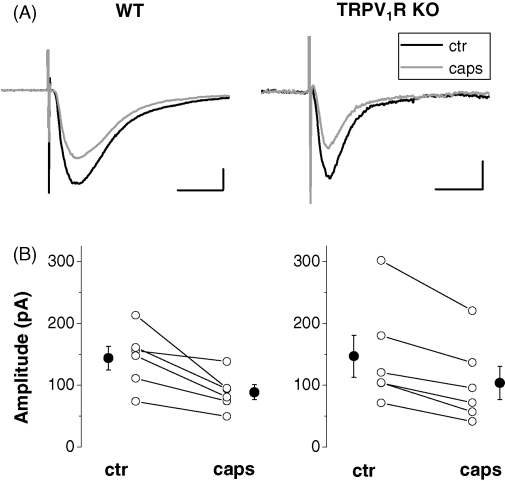
The suppression of excitatory postsynaptic currents by capsaicin in dentate granule cells recorded in wild type (WT) or TRPV_1_R knockout (KO) mice. (A): Representative averaged recordings of six to eight consecutive EPSCs taken before (black) and after application of 10 μM capsaicin (gray) in wild type mice (WT) or in TRPV_1_R knockouts. Scale bars are 25 pA and 10 ms. (B): Individual values (open circles) and averaged data (solid circles) for the capsaicin-induced suppression of EPSCs in wild type and TRPV_1_R knockout mice are shown.

The effects of the prototypic TRPV_1_R agonist capsaicin on EPSCs evoked by focal stimulation of fibers in the stratum moleculare were measured in dentate granule cells of wild type mice and TRPV_1_R knockouts. Similar to what we found earlier (Hájos and Freund, 2002), bath application of 10 μM capsaicin significantly reduced the amplitude of EPSCs (by 36.6 ± 6.1% of control) in wild type mice (control: 143.8 ± 19.3 pA; capsaicin: 88.6 ± 12.1 pA; *n* = 6; *p* < 0.02; Fig. 2A and B). In TRPV_1_R knockout mice, a similar significant reduction was observed after capsaicin application, the amplitude of EPSCs was suppressed by 31.6 ± 4.1% of control (control: 146.9 ± 34.2 pA; capsaicin: 103.8 ± 26.9 pA; *n* = 6; *p* < 0.02; Fig. 2A and B). The inhibitory effect of capsaicin on the amplitude of EPSCs was indistinguishable in wild type mice and TRPV_1_R knockouts (*p* > 0.1).

**Fig. 3:**
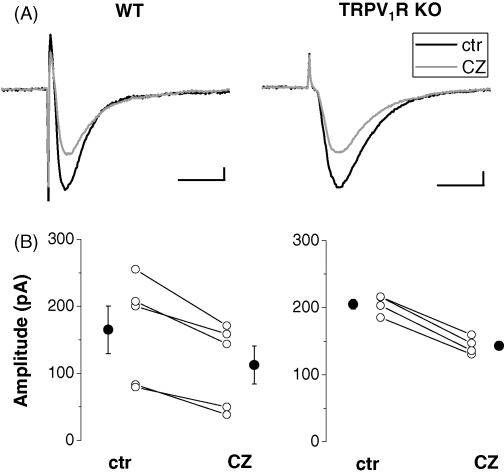
The reduction of excitatory postsynaptic currents by capsazepine (CZ) in dentate granule cells recorded in wild type (WT) or TRPV_1_R knockout (KO) mice. (A): Representative averaged recordings of 8–10 consecutive EPSCs taken before (black) and after application of 10 μM capsazepine (gray) in wild type mice (WT) or in TRPV_1_R knockouts. Scale bars are 25 pA and 10 ms. (B): Individual values (open circles) and averaged data (solid circles) for the capsazepine-induced suppression of EPSCs in wild type and TRPV_1_R knockout mice are shown.

Next, we tested the effect of 10 μM capsazepine, a TRPV_1_R antagonist, on excitatory synapses. We found that bath application of this drug also significantly reduced the EPSC amplitude (by 34.9 ± 5.4% of control) in wild type animals (control: 165.1 ± 35.5 pA, capsazepine: 112.4 ± 28.2 pA; *n* = 5; *p* < 0.05; Fig. 3A and B). When we co-applied 10 μM capsaicin together with 10 μM capsazepine, the amplitude of evoked EPSCs was similarly decreased (by 33.3 ± 9.4% of control; control: 149.5 ± 21.2 pA; capsaicin + capsazepine: 99.6 ± 19.6 pA; *n* = 4; *p* < 0.05). In TRPV_1_R knockouts, suppression of the EPSC amplitude was comparable to that seen in wild type mice (i.e., by 30.2 ± 1.5% of control after capsazepine treatment; control: 205.2 ± 7.3 pA; capsazepine: 143.2 ± 6.4 pA; *n* = 4; *p* < 0.05; Fig. 3A and B). Similarly, the treatment of slices with a mixture of capsaicin and capsazepine reduced the EPSC amplitude by 38.1 ± 6.2% of control (control: 211.8 ± 4.4 pA, capsaicin + capsazepine: 131.3 ± 13.5 pA; *n* = 3; *p* < 0.05), just like in the wild types. These results suggest that capsazepine, as well as capsaicin alone can reduce the amplitude of EPSCs independent of TRPV_1_Rs, and their effects are not additive.

By a comparison of the paired-pulse ratios of evoked EPSCs, we next examined whether capsaicin and capsazepine affect excitatory synapses presynaptically or postsynaptically. If glutamate release is altered, then the paired-pulse ratio should change. If the reduction in EPSC amplitude is not accompanied by changes in the paired-pulse ratio, then the conductivity of glutamate receptors should be modified by the drug treatment. Therefore, we first investigated the effect of capsaicin on paired-pulse ratio in wild type mice and TRPV_1_R knockouts. After drug application, the ratio significantly increased to 121.3 ± 3.2% of control in wild types and to 132.5 ± 9.9% of control in knockouts (*n* = 6 each, *p* < 0.02). Comparable to these findings, capsazepine treatment also caused a significant increase in the paired-pulse ratio both in wild types (129.7 ± 10.1%; *n* = 5; *p* < 0.05) and knockouts (126.4 ± 8.8; *n* = 4; *p* < 0.05). Thus, the effects of both capsaicin and capsazepine appear to be presynaptic, reducing glutamate release from excitatory terminals both in wild type and TRPV_1_R knockout mice.

**Fig. 4:**
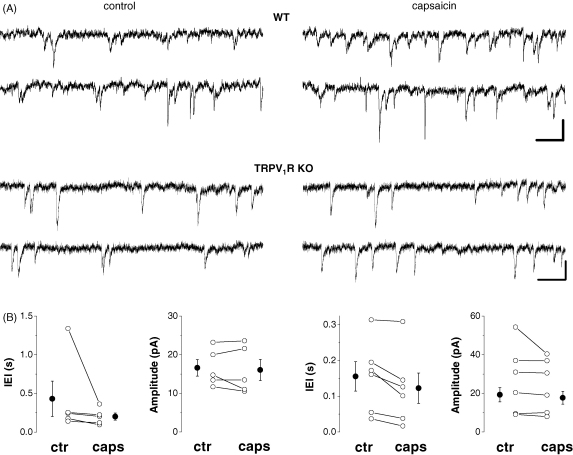
The occurrence of spontaneous EPSCs in dentate granule cells is increased by capsaicin both in wild type and TRPV_1_R knockout mice. (A): Representative raw recordings before and after bath application of 10 μM capsaicin. Scale bars are 10 pA and 100 ms. (B): Median of individual experiments (open circles) and averaged values (filled circles) for the inter-event interval (IEI) and the amplitude of spontaneous EPSCs in control and after drug application are shown. Capsaicin induced a significant reduction in the inter-event intervals of synaptic events (i.e., increased the frequency) without changing their amplitude both in wild type and TRPV_1_R knockout mice.

In further experiments, we investigated how capsaicin alters the properties of spontaneous EPSCs (sEPSCs). In wild type mice, bath application of capsaicin significantly increased the occurrence of spontaneous events (i.e., reduced the inter-event interval by 32.5 ± 11.8% of control, control: 0.43 ± 0.23 s; capsaicin: 0.2 ± 0.05 s; *n* = 5; *p* < 0.04, Fig. 4A and B) without changing their amplitude (control: 16.6 ± 2.1 pA; capsaicin: 16.1 ± 2.7 pA; *n* = 5; *p* > 0.1; Fig. 4A and B). Similarly, capsaicin also elevated the frequency of sEPSCs in TRPV_1_R knockouts, since the inter-event interval was reduced by 29.3 ± 7.4% of control (control: 0.16 ± 0.04 s; capsaicin: 0.12 ± 0.04 s; *n* = 6; *p* < 0.02; Fig. 4A and B). Similar to those observed in wild types, the amplitude of synaptic events did not change (control: 19.2 ± 3.6 pA; capsaicin: 17.7 ± 3.3 pA; *n* = 6; *p* > 0.1; Fig. 4A and B). The comparison of the decrease in the inter-event interval of sEPSCs between wild type mice and TRPV_1_R knockouts showed no difference (*p* > 0.1). These results provided further evidence that capsaicin affected synaptic glutamate release in wild type and TRPV_1_R knockout mice to a similar degree, in a similar manner.

## 4 Discussion

Electrophysiological data presented here strongly suggest that capsaicin actions on excitatory synaptic transmission are not mediated by TRPV_1_Rs, at least in the dentate gyrus. Our previous observations already raised this possibility (Hájos and Freund, 2002), as we have shown that the suppression of the amplitude of EPSCs after the second application of capsaicin was indistinguishable from that seen after the first application. This observation was not consistent with the known desensitization properties of TRPV_1_Rs upon repeated capsaicin application (Dray et al., 1989; Docherty et al., 1991; Caterina et al., 1997). Our results seem to contradict those pharmacological data, where the effect of capsaicin on synaptic transmission has been found to be fully blocked by antagonists specific for TRPV_1_Rs (e.g., capsazepine or iodo-resiniferatoxin) (Al-Hayani et al., 2001; Marinelli et al., 2003). Here we found that, in adult mice, capsazepine also effectively reduced the amplitude of EPSCs, similar to that seen after capsaicin application. In line with these data, a study by Kofalvi et al. (2003) has shown that glutamate release from synaptosomes prepared from the hippocampus of adult rats could be significantly suppressed by capsazepine. These findings are in contrast with our published results (Hájos and Freund, 2002), where capsazepine could antagonize the effect of capsaicin on EPSC amplitude recorded in slices from rats of P15-22. We repeated the experiments with capsazepine in young rats and found that this drug on its own could substantially enhance the amplitude of EPSCs (unpublished observations), which is in sharp contrast observed in adult animals (present study; Kofalvi et al., 2003). Thus, it seems likely that during development the molecular target of capsazepine changes its effect on synaptic transmission, or the binding site(s) of capsazepine might alter. We therefore propose that, in the hippocampus of young rats, the suppression of EPSC amplitude by capsaicin is counterbalanced by the enhancement caused by capsazepine, therefore no reduction in glutamate release can be observed.

As to the presynaptic mechanism of capsaicin actions, a recent study showed that iodo-resiniferatoxin as well as capsaicin (both applied in μM concentrations) could markedly reduce the high K^+^-induced Ca^2+^ entry (Kofalvi et al., 2006). Since transmitter release is highly sensitive to Ca^2+^ entry, one might assume that glutamate release from excitatory terminals in the dentate gyrus could be affected with a similar mechanism. In the present study we found that capsaicin, capsazepine, or co-application of the two, reduce the amplitude of EPSCs to a similar extent, suggesting that capsazepine might also decrease Ca^2+^ entry at the same site, where capsaicin acts.

Data from other laboratories, as well as our own results, showed that capsaicin could reduce the amplitude of evoked EPSCs, while it increased the frequency of spontaneous EPSCs without changing their amplitude (Hájos and Freund, 2002; Marinelli et al., 2002, 2003). These unconventional effects seem to suggest that the target molecule of capsaicin regulating glutamatergic transmission could be located at the presynaptic axon terminals, an assumption that is also supported by the observed increase in the paired-pulse ratio (present study). If capsaicin modulated postsynaptic glutamate receptors, the amplitude of sEPSCs should have also been altered, which was not the case (present study; Marinelli et al., 2003). The question arises how capsaicin could reduce the amplitude of evoked EPSCs, while in the same time increases the frequency of sEPSCs. One explanation might be that if capsaicin triggers release of retrograde messengers from postsynaptic neurons that directly promote fusion of vesicles irrespective of Ca^2+^ concentration within the terminals, this could enhance the action potential-independent glutamate release (i.e., the majority of sEPSCs under our circumstances). Although TRPV_1_Rs have been shown to be located in the dendrites and somata of hippocampal principal cells (Cristino et al., 2006), these receptors are unlikely to be involved in the enhancement of sEPSC frequency, since the same effect was observed in the TRPV_1_R knockout mice. Further studies should clarify the possible mechanisms underlying the capscaicin-induced changes in excitatory neurotransmission.

In summary, our data presented here suggest that TRPV_1_Rs may not be the target molecules for capsaicin regulating glutamatergic synapses in the hippocampal network, although they might have a function during pathological conditions like fever or osmotic changes in extracellular space (Caterina et al., 1997; Gavva et al., 2007; Liu et al., 2007).
